# Overexpression of a Novel *ROP* Gene from the Banana (*MaROP5g*) Confers Increased Salt Stress Tolerance

**DOI:** 10.3390/ijms19103108

**Published:** 2018-10-11

**Authors:** Hongxia Miao, Peiguang Sun, Juhua Liu, Jingyi Wang, Biyu Xu, Zhiqiang Jin

**Affiliations:** 1 Key Laboratory of Biology and Genetic Resources of Tropical Crops, Ministry of Agriculture, Institute of Tropical Bioscience and Biotechnology, Chinese Academy of Tropical Agricultural Sciences, Xueyuan Road 4, Haikou 571101, China; miaohongxia@itbb.org.cn (H.M.); liujuhua@itbb.org.cn (J.L.); wangjingyi@itbb.org.cn (J.W.); 2 Key Laboratory of Genetic Improvement of Bananas, Hainan Province, Haikou Experimental Station, Chinese Academy of Tropical Agricultural Sciences, Xueyuan Road 4, Haikou 570102, China; sunpeiguang@catas.cn

**Keywords:** banana (*Musa acuminata* L.), ROP, genome-wide identification, abiotic stress, salt stress, *MaROP5g*

## Abstract

Rho-like GTPases from plants (ROPs) are plant-specific molecular switches that are crucial for plant survival when subjected to abiotic stress. We identified and characterized 17 novel ROP proteins from *Musa acuminata* (MaROPs) using genomic techniques. The identified MaROPs fell into three of the four previously described ROP groups (Groups II–IV), with MaROPs in each group having similar genetic structures and conserved motifs. Our transcriptomic analysis showed that the two banana genotypes tested, Fen Jiao and BaXi Jiao, had similar responses to abiotic stress: Six genes (*MaROP-3b*, *-5a*, *-5c*, *-5f*, *-5g*, and *-6*) were highly expressed in response to cold, salt, and drought stress conditions in both genotypes. Of these, *MaROP5g* was most highly expressed in response to salt stress. Co-localization experiments showed that the MaROP5g protein was localized at the plasma membrane. When subjected to salt stress, transgenic *Arabidopsis thaliana* overexpressing *MaROP5g* had longer primary roots and increased survival rates compared to wild-type *A. thaliana*. The increased salt tolerance conferred by *MaROP5g* might be related to reduced membrane injury and the increased cytosolic K^+^/Na^+^ ratio and Ca^2+^ concentration in the transgenic plants as compared to wild-type. The increased expression of salt overly sensitive (SOS)-pathway genes and calcium-signaling pathway genes in *MaROP5g*-overexpressing *A. thaliana* reflected the enhanced tolerance to salt stress by the transgenic lines in comparison to wild-type. Collectively, our results suggested that abiotic stress tolerance in banana plants might be regulated by multiple *MaROPs*, and that *MaROP5g* might enhance salt tolerance by increasing root length, improving membrane injury and ion distribution.

## 1. Introduction

Small GTPases (GTP)-binding proteins, present in a wide variety of eukaryotes, are the central regulators of numerous signal transduction processes [1,2]. These proteins are structurally classified into at least five families, including Rat sarcoma (RAS), Ras homolog (RHO), Rat brain (RAB), RAS-related nuclear (RAN), and adenosine diphosphate (ADP) ribosylation factor (ARF) [1,2,3]. In all reported eukaryotes, RAS and RHO families are signaling switches, whereas these proteins in other families are primarily involved in the regulation of vesicle and large molecule movement [1,2]. However, higher plants have a unique RHO subfamily of small GTP-binding proteins known as ROPs (Rho-like GTPases from plants) [4,5]. These proteins are also known as RAS-related C3 botulinum toxin substrates (RACs), due to their sequence similarity to Rac GTPases [6].

ROPs are plant-specific molecular switches that regulate intracellular signaling pathways by cycling between an active form and an inactive, guanosine diphosphate (GDP)-bound form. Biological activities associated with ROPs are diverse, which include polar growth, development, environmental stress responses, and host-pathogen interactions [7,8,9,10,11,12,13]. Since the first *ROP* gene was isolated from peas [14], multiple *ROPs*, with typical RhoGEF domains and molecular masses between 21 and 24 kDa, have been described in numerous plant species: 11 in *Arabidopsis thaliana* [15], 9 in *Zea mays* [16], 11 in *Brassica napus* [17], 7 in *Vitis vinifera* [9], 7 in *Oryza sativa* [18], 7 in *Medicago truncatula* [7], 6 in *Nicotiana tabacum* [7], 5 in *Hevea brasiliensis* [19], and 9 in *Solanum lycopersicum* [20]. *ROPs* can be classified into four groups (I–IV) based on their molecular structure and motif conservation [5,7,16].

ROP expression levels and biological functions are affected by various abiotic stressors. When exposed to cold, the transcriptional expression of apple *ROP* increases, leading to a decrease in the concentrations of ethylene and reactive oxygen species in the fruits [21]. In *A. thaliana*, *ROP11* expression affected seed germination, seedling growth, stomatal closure, abscisic acid (ABA)-mediated responses, and drought stress responses [22]. The overexpression of *ROP1* in tobacco increased salt sensitivity in response to salt stress by increasing H_2_O_2_ production [23]. The Na^+^/K^+^ ratio in transgenic *A. thaliana* expressing the *Medicago falcate* small GTPase gene (*MfARL1*) was lower than that in wild-type (WT) *A. thaliana*; the transgenic plants consequently had an increased tolerance for salt stress [24]. Knock out of the *A. thaliana* ROP effector (RIC1) increased the survival rate of plants under salt stress by improving the reassembly of depolymerized microtubules [25]. Taken together, these studies have revealed the many important roles of ROPs in the regulation of plant response to abiotic stresses.

The banana (*Musa acuminata*) is one of the most intensively produced and globally important fruit crops [26]. As a large monocotyledonous herbaceous annual, banana plants are frequently harmed or destroyed by various abiotic stress conditions during growth and development [27]. In particular, saline soil is a major abiotic stressor which limits banana cultivation worldwide [28,29]. Genome-wide identification of genes involved in the resistance of banana plants to cold, drought, and salt stress increases our knowledge of plant mechanisms for environmental stress tolerance, while the functional identification of relevant genes acts as a framework for future genetic studies focused on increasing the resistance of the banana plant to these stressors [30,31,32,33]. However, genome-wide investigations of the *ROP* gene family, and thus an integrated assessment of the potential functions of this important molecular switch, are still lacking in banana.

To address this information gap, we identified *ROPs* genome-wide in *M. acuminata,* known as *MaROPs*, and analyzed their phylogenetic relationships, gene structures, protein motifs, and expression changes in response to a number of abiotic stressors, including cold, drought, and salt. More importantly, we noted that the expression of the *MaROP5g* gene was associated with salt stress in banana. The overexpression of *MaROP5g* in *A. thaliana* conferred increased salt tolerance by lengthening roots, improving recovery of membrane injury and ion distributions. This comprehensive study of *MaROPs* in *M. acuminata* enhances our understanding of *ROPs* in response to abiotic stress conditions in banana plants, and provides a foundation for future studies aiming to improve the abiotic stress resistance of crop plants, especially with respect to salt stress.

## 2. Results

### 2.1. Identification and Phylogenetic Analysis of Banana MaROP Genes

We used the basic local alignment search tool (BLAST) and the hidden Markov models (HMM) to identify MaROPs with typical RhoGEF domains (PF00621) in the *M. acuminata* genome, using the sequences of AtROPs and OsROPs as queries [34,35]. We identified 17 MaROPs in the *M. acuminata* genome, and designated these MaROP-2a, -2b, -2c, -3a, -3b, -4, -5a, -5b, -5c, -5d, -5e, -5f, -5h, -5g, -6, -7a, and -7b, following the nomenclature of their respective orthologous proteins in *O. sativa*. The 17 predicted MaROP proteins ranged from 195 amino acid residues (MaROP5d) to 214 amino acid residues (MaROP4), with relative molecular masses between 21.297 kDa (MaROP5d) and 23.784 kDa (MaROP3a), and isoelectric points between 8.61 and 9.43 (Table S1).

**Figure 1:**
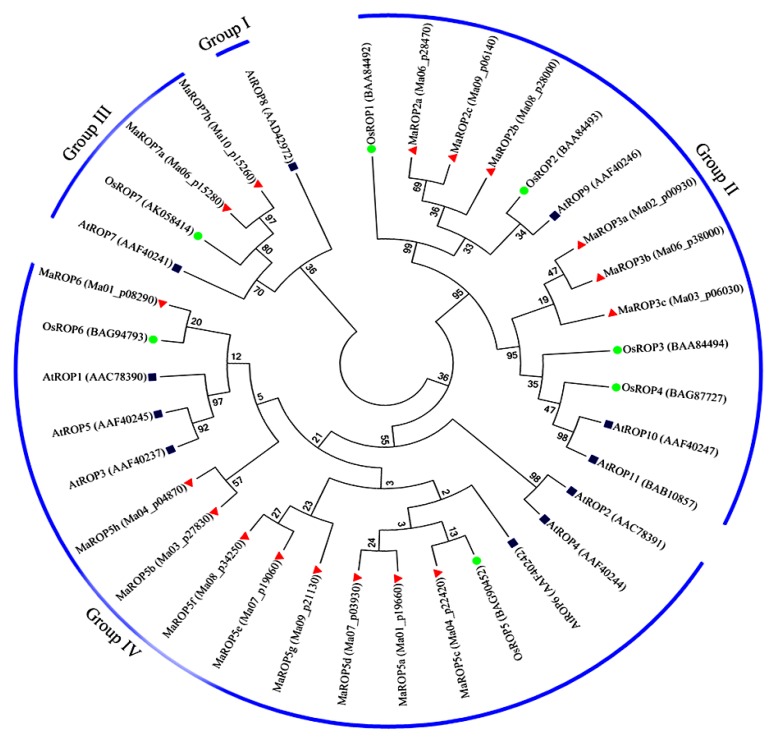
Phylogenetic analysis of Rho-like GTPases from plants (ROPs) from *A. thaliana*, rice, and bananas. The maximum-likelihood phylogenetic tree was drawn with MEGA5.2, using 1000 bootstraps. Four subgroups were identified (Groups I–IV). Circles, squares, and triangles represent ROP proteins from rice, *A. thaliana*, and bananas, respectively.

To investigate the evolutionary relationships among ROP proteins, we constructed a maximum-likelihood (ML) phylogenetic tree based on our multiple sequence alignment of 17 ROP proteins from *M. acuminata*, 11 from *A. thaliana*, and 7 from *O. sativa*. The MaROP proteins fell into 3 distinct groups (Figure 1): Group II contained 6 MaROPs (MaROP-2a, -2b, -2c, -3a, -3b, and -4), 3 AtROPs (AtROP-9, -10, and -11), and 4 OsROPs (OsAPL-1, -2, -3, and -4); Group III contained MaROP-7a, -7b, AtROP7, and OsROP7; and Group IV contained 9 MaROPs (MaROP-5a, -5b, -5c, -5d, -5e, -5f, -5g, -5h, and -6), 6 AtROPs (AtROP-1, -2, -3, -4, -5, and -6), and 3 OsROPs (OsROP-2, -5, and -6). Group I containing AtROP8 served as an outgroup to the phylogenetic analysis.

### 2.2. Gene Structure and Conserved Motif Analysis of Banana MaROP Genes

**Figure 2:**
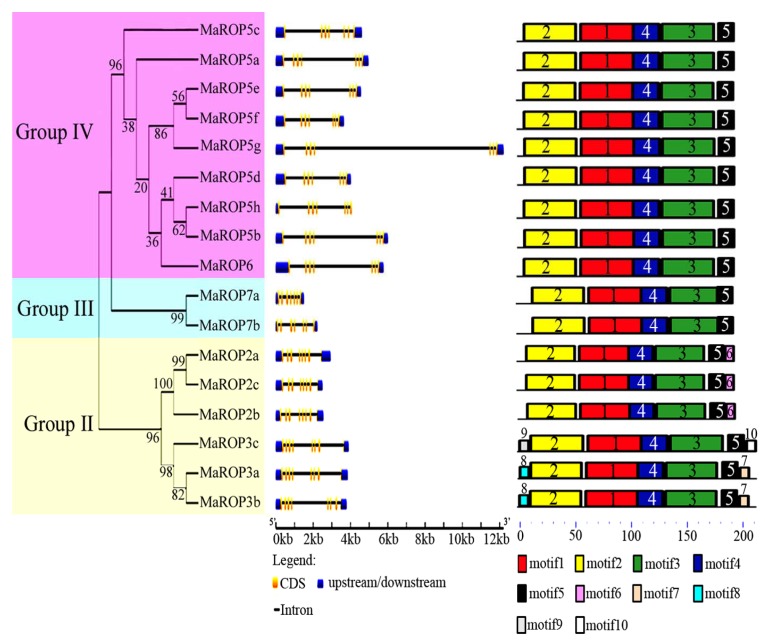
Phylogenetic, gene structure, and motif analyses of banana *Musa acuminata* ROP (MaROP) proteins. MaROPs were classified into Groups II–IV based on their phylogenetic relationships. Exon–intron structure analyses were performed with the Gene Structure Display Server (GSDS). Blue boxes indicate upstream/downstream; yellow boxes indicate exons; black lines indicate introns. All of the proteins were identified using the Multiple EM for Motif Elicitation (MEME) database, using the complete predicted amino acid sequences of each MaROP. Motifs 1–4 were annotated as a RhoGEF domain.

Evolutionary analysis supported the classification of the 17 *MaROP* genes into three distinct groups (Groups II–IV), which is consistent with their exon–intron structural divergence within families (Figure 2)*.* Our analysis of the exon–intron structure using the Gene Structure Display Server showed that the *MaROP* genes contained 8 exons in Group II, 7 exons in Group III, and 6–7 exons in Group IV, suggesting the conservation of the exon–intron structures of *MaROPs* within the same group.

To explore MaROPs structural diversity and potential functionality, we analyzed the conserved motifs of the identified MaROPs and predicted their functional annotations. We identified 10 conserved motifs across the 17 MaROP proteins with Multiple Em (Motif Elicitation), which are annotated with InterPro (Figure 2; Table S2). Motifs 1–4 were annotated as a RhoGEF domain (PF00621), the characteristic domain of the ROP protein family. Motifs 1–5 were found across all of the 17 MaROPs, while motif 6 was only present in MaROP-2a, -2b, and -2c; motifs 6–8 were found in MaROP-3a and -3b; and motifs 9 and 10 were found in MaROP4. It is probable that this pattern of conservation and variation across the motifs reflected the evolutionary relatedness and functional divergence of the 17 MaROPs.

### 2.3. Expression Analysis of MaROP Genes in Response to Cold, Salt, and Osmotic Stresses

**Figure 3:**
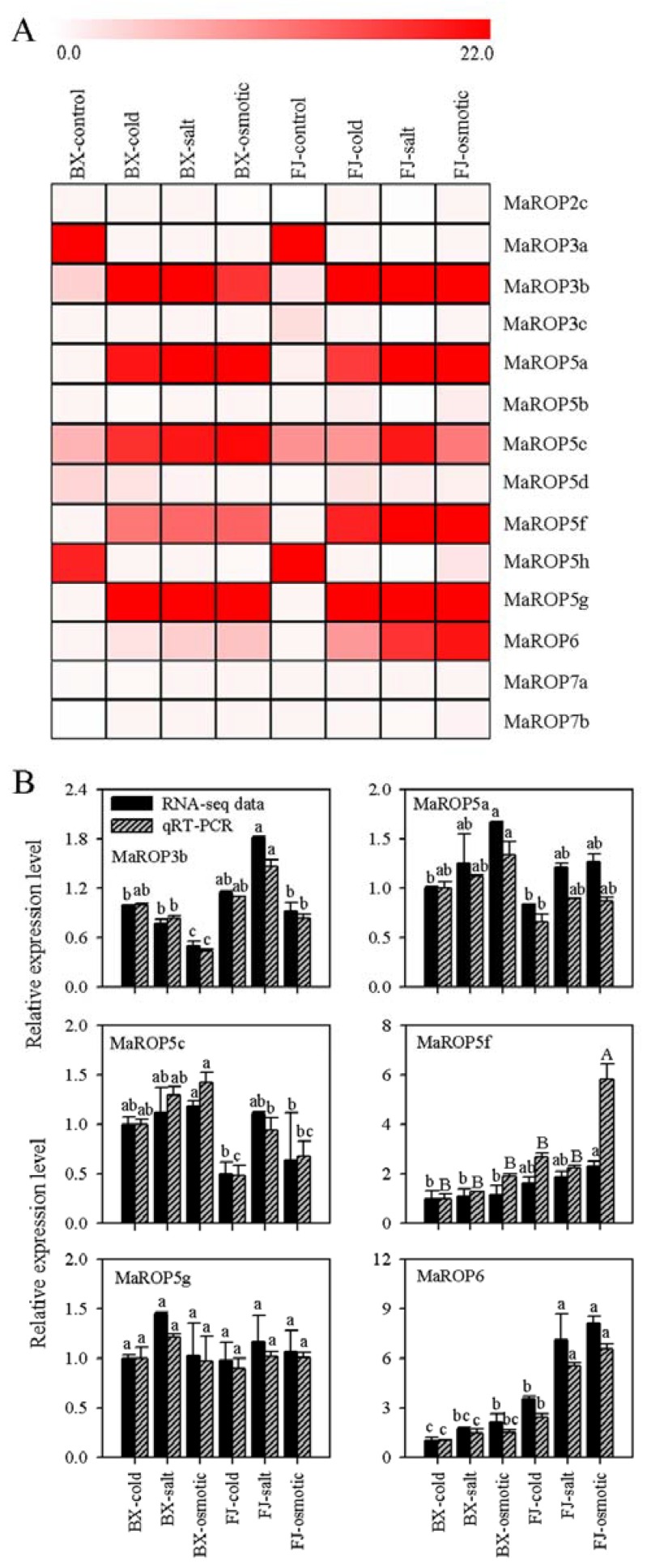
Differential expression of Banana *MaROPs* in response to cold, salt, and osmotic stresses in BaXi Jiao (BX; *M. acuminata* cv. Cavendish; AAA group) and Fen Jiao (FJ; *M. acuminata*; group AAB) banana varieties, as determined with transcriptomic analysis and qRT-PCR. (**A**) Heat map clusters were created based on the fragments per kilobase of exon per million fragments mapped (FPKM) value of the *MaROPs*. Magnitude of differences in gene expression is indicated with a one-color scheme. (**B**) Data are presented as means ± standard deviations of *n* = 3 biological replicates. Different lowercase letters above bars indicate significant differences at *p* < 0.05, and different uppercase letters above bars indicate extremely significant differences at *p* < 0.01, using Duncan’s multiple range tests.

To investigate the response of the *MaROP* genes in response to different abiotic stressors, we analyzed the *MaROP* expression in banana leaves following exposure to cold, salt, and osmotic stress conditions (Figure 3A; Table S3). Compared to the control, significant differences in the expression of 14 *MaROP* genes (82%) were detected following the exposure to abiotic stress treatments (Figure 3A; Table S3). In BaXi Jiao (BX), the expression levels of *MaROP-3b*, *-5a*, *-5c*, *-5f*, *-5g*, and *-6* were significantly upregulated, as indicated by the fragments per kilobase of exon per million fragments mapped (FPKM) value, which is higher than 2.0 by all three of the abiotic stressors. *MaROP5d* was upregulated by cold treatment only (FPKM > 2.0), and *MaROP2c* was downregulated by osmotic treatment only (FPKM < 0.5). In Fen Jiao (FJ), *MaROP-3b*, *-5a*, *-5c*, *-5f*, *-5g*, *-6* were significantly expressed (FPKM > 8.9) in the presence of abiotic stressors. Under osmotic treatment, *MaROP6* was upregulated (FPKM > 2.3) in FJ, but maintained a low level of expression in BX (FPKM < 0.66). In addition, *MaROP-3a* and *-5h* are significantly downregulated in the presence of stress in BX and FJ, compared to control. *MaROP5g* had a higher level of expression (FPKM > 24) in response to salt stress than any of the other *ROPs* across both the BX and FJ genotypes, implying *MaROP5g* might play an important role in the regulation of salt stress tolerance in banana plants.

### 2.4. Validation of Differential Expression of Six MaROP Genes by Quantitative Real-Time Polymerase Chain Reaction (qRT-PCR) Analysis

Our RNA-seq analysis indicated that *MaROP-3b*, *-5a*, *-5c*, *-5f*, *-5g*, and *-6* showed significant expressions by abiotic stressors. Such a feature of these six genes was further verified by quantitative real-time polymerase chain reaction (qRT-PCR) analysis. After normalization, all the examined *MaROPs*, with the exception of *MaROP3b* in FJ-salt and *MaROP5c* in FJ-salt, were well-correlated and generally consistent with our RNA-seq analyses (*r* = 0.8789–0.9992; Figure 3B; Table S4), indicating the reliability of our transcriptomic results in both banana varieties. Of particular interest was *MaROP5g*, which showed high expression following salt stress treatment compared to other *MaROP* genes, as determined by both RNA-seq and qRT-PCR experiments.

### 2.5. Full-Length cDNA, Subcellular Localization, and Expression Pattern of MaROP5g under Salt Stress

Based on the results of the RNA-seq and qRT-PCR analyses, we used PCR to amplify the full-length cDNA of *MaROP5g* from banana roots. The full-length *MaROP5g*cDNA had a 591 bp open reading frame (ORF), encoding 196 amino acids. The predicted MaROP5g protein had a typical RhoGEF domain and several additional characteristics of the ROP protein family (Figure S1; Table S2).

**Figure 4:**
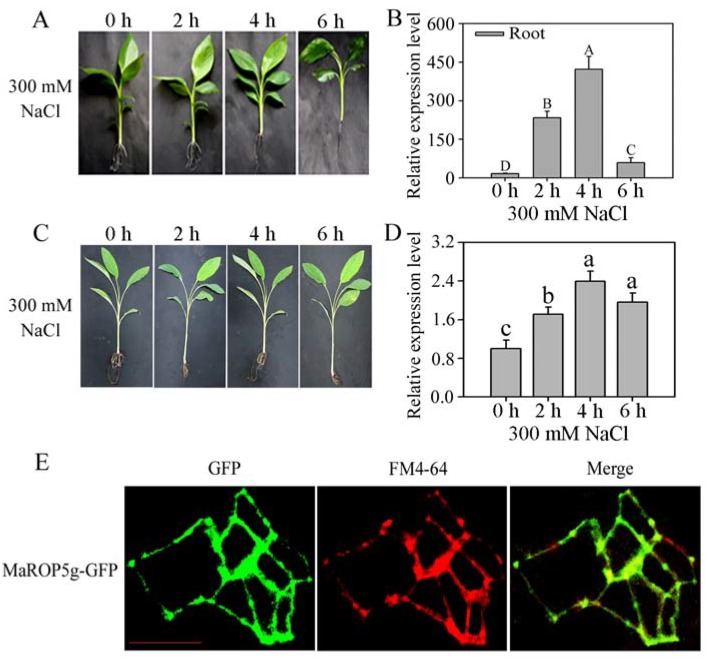
*MaROP5g* expression analyses in both banana varieties roots after different periods of exposure to salt stress and subcellular localization. (**A**) Phenotypes of BX roots exposed to salt stress; (**B**) expression of *MaROP5g* in BX roots exposed to salt stress; (**C**) phenotypes of FJ roots exposed to salt stress; (**D**) expression of *MaROP5g* in FJ roots exposed to salt stress. Data are presented as means ± standard deviations of *n* = 3 biological replicates. Different lowercase letters above bars indicate significant differences at *p* < 0.05, and different uppercase letters above bars indicate extremely significant differences at *p* < 0.01, using Duncan’s multiple range tests. (**E**) MaROP5g subcellular localization. GFP fluorescence is green, and FM4-64 is red. Merge was created by merging the GFP and FM4-64 fluorescent images. Scale bars = 10 μm.

We measured the transcriptional response of *MaROP5g* in BX and FJ plant roots to salt stress. Compared to 0 h (no stress condition), the roots of the BX plants became black following 6 h of salt stress treatment (Figure 4A). However, there was no discernible phenotypic change in the roots of FJ plants under the same treatment (Figure 4C). The expression of *MaROP5g* quickly increased from 0 h, reached the maximum level at 4 h, and then gradually decreased at 6 h (Figure 4B). The expression pattern of *MaROP5g* was similar between FJ and BX (Figure 4D), but *MaROP5g* showed lower expression in FJ compared to BX under salt stress. These results suggested that the regulation of *MaROP5g* expression by salt treatment was genotype-dependent, as the roots of the two tested banana genotypes may have variable sensitivity to salt stress treatments. BX showed more sensitivity than FJ under salt stress treatment.

To localize the MaROP5g protein in the cell, we introduced the *MaROP5g* ORF into a pCAMBIA1304-GFP vector upstream of the *GFP* gene to create a MaROP5g-GFP fusion construct, which was used to transform *A. thaliana*. Co-localization experiments showed that the MaROP5g-GFP (green fluorescent protein) fusion protein was localized to the FM4-64-labeled plasma membrane in *A. thaliana* root tips (Figure 4E).

### 2.6. MaROP5g Overexpression Enhances Tolerance to Salt Stress

To investigate the role of *MaROP5g* in response to salt stress, *MaROP5g* was introduced into the pCAMBIA1304 vector under the control of the 35S promoter. After a floral-dip transformation of *A. thaliana*, we analyzed three transgenic lines with single-copy transgene (R3, R8, and R42) from the T3 generation, selected through genomic DNA Southern blot analysis (Figure S2A). The expression level of *MaROP5g* in three transgenic lines was 89–109 folds compared to WT and empty vector (VC), as revealed by qRT-PCR analysis (Figure S2B).

**Figure 5:**
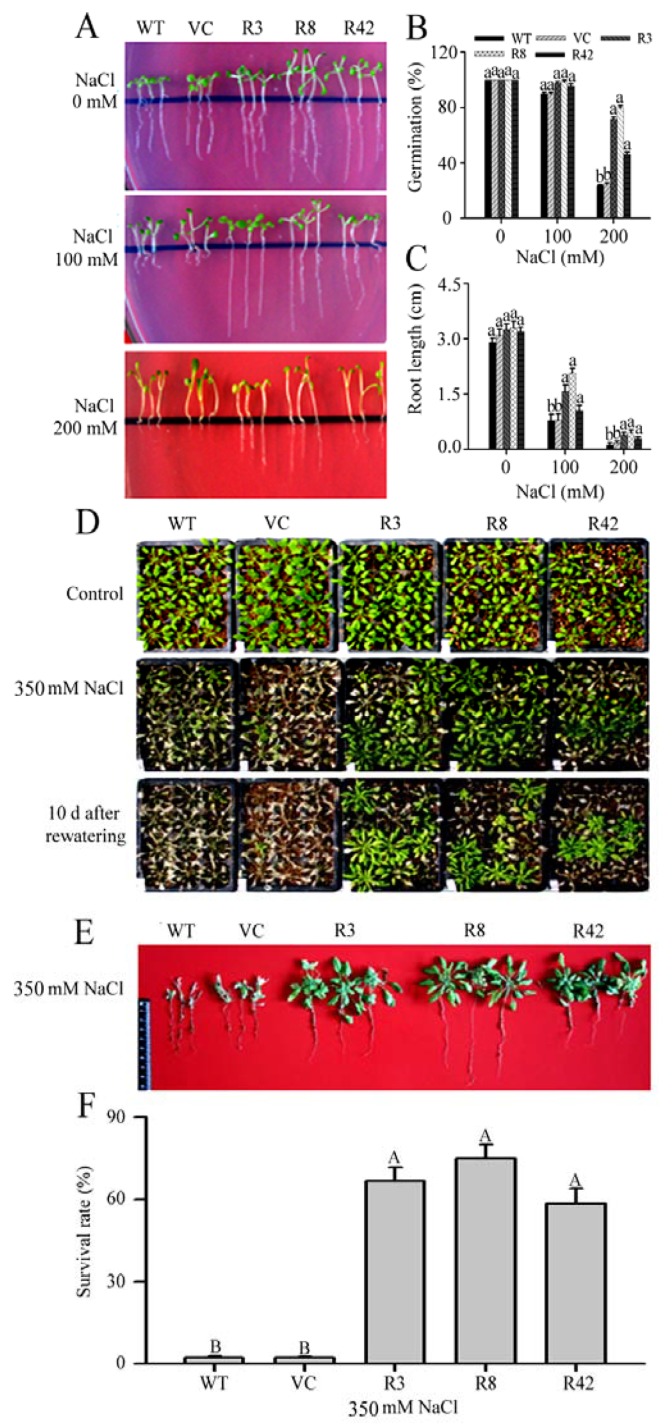
Comparison of wild-type and *A. thaliana* overexpressing *MaROP5g* under standard growing conditions (control) and different salt stress treatments. (**A**) Phenotypes of WT and transgenic lines under control and salt conditions; (**B**) Germination of WT and transgenic lines under control and salt conditions; (**C**) Root length of WT and transgenic lines under control and salt conditions; (**D**,**E**) Phenotypes of WT and transgenic mature plants under control or salt conditions or after rewatering; (**F**) Survival rates of WT and transgenic mature plants under control or salt conditions. WT: wild-type. VC: vector. R3, R8, R42: *MaROP5g* transgenic plants. ANOVA was used to compare the significance of differences, using Dunnett’s tests in the comparison between WT and each overexpression lines. Data are presented as means ± standard deviations of *n* = 3 biological replicates. Different lowercase letters above bars indicate significant differences at *p* < 0.05, different uppercase letters above bars indicate extremely significant differences at *p* < 0.01.

Under no-salt (control) and high-salt conditions, the seed germination rate and root growth were greater in the transgenic seedlings as compared to those in the WT seedlings. After salt treatments ranging from 100 to 200 mM, the transgenic seedlings had grown longer primary roots, as compared to WT seedlings (Figure 5A–C). Furthermore, when adult *A. thaliana* growing in soil were treated with 350 mM salt daily for 15 days, the transgenic lines grew better (Figure 5D,E) and were more likely to survive (Figure 5F), as compared to those of WT. Thus, transgenic *A. thaliana* lines overexpressing *MaROP5g* were more tolerant to salt stress than those of WT.

### 2.7. MaROP5g Overexpression Reduced Malonaldehyde (MDA) Content and Ion Leakage (IL), Increased Ca^2+^ and K^+^/Na^+^ Ratio under Salt Stress

**Figure 6:**
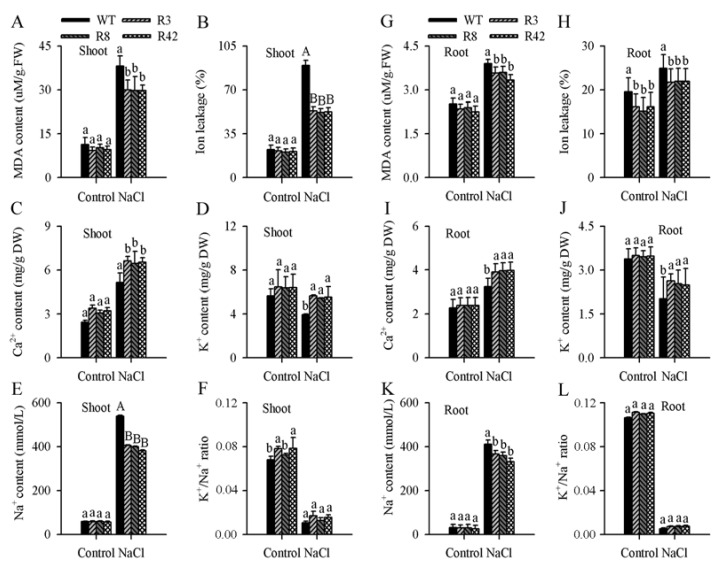
Physiological analyses and ion concentration in roots from wild-type and *A. thaliana* overexpressing *MaROP5g*. (**A**–**F**) Malonaldehyde content, ion leakage, Ca^2+^ concentration, K^+^ concentration, Na^+^ ion concentration, and K^+^/Na^+^ ratio of wild-type and transgenic shoots under normal conditions and salt treatment. (**G**–**L**) Malonaldehyde content, ion leakage, Ca^2+^ concentration, K^+^ concentration, Na^+^ ion concentration, and K^+^/Na^+^ ratio of wild-type and transgenic roots under normal conditions and salt treatment. WT: Wild-type. R3, R8, R42: *MaROP5g* transgenic plants. ANOVA was used to compare the significance of differences, using Student’s *t* tests in the comparison between control and NaCl treatment. Data are presented as means ± standard deviations of *n* = 3 biological replicates. Different lowercase letters above bars indicate significant differences at *p* < 0.05, and different uppercase letters above bars indicate extremely significant differences at *p* < 0.01.

Malonaldehyde (MDA) is usually employed as an index of oxidative damages in plants [36]. Ion leakage (IL) is an important indicator of membrane injury [36]. To investigate whether *MaROP5g* expression influences MDA and IL content, we measured MDA and IL content in the shoots and roots of transgenic lines and WT plants, following high-salt treatments vs. no-salt control (Figure 6). Following high-salt treatment, MDA content was lower in the shoots and roots of the transgenic plants as compared to those of WT (Figure 6A–G). Similarly, IL value was lower in the shoots and roots of transgenic plants as compared to those of WT under high salt treatment (Figure 6B–H). Taken together, these results suggest that the overexpression of *MaROP5g*in transgenic *A. thaliana* plants may have prevented or reduced membrane injury under salt stress as compared to WT.

Under highly saline conditions, plant cells survive by retaining a high cytosolic Ca^2+^ concentration and a high K^+^/Na^+^ ratio [37,38]. Under high-salt treatment, the concentrations of Ca^2+^ and K^+^ in the shoots or roots of transgenic *A. thaliana* plants were greater (Figure 6C,D), while the Na^+^ concentration was lower in the shoots or roots of transgenic *A. thaliana* plants as compared to those of WT (Figure 6E). Therefore, the shoots or roots of the transgenic lines maintained higher K^+^/Na^+^ ratios than did those of the WT plants during salt treatment (Figure 6F). These results suggested that the overexpression of *MaROP5g* in plants subjected to salt stress increased cellular Ca^2+^ and K^+^ accumulation, decreased cellular Na^+^ accumulation, and improved the K^+^/Na^+^ ratio.

### 2.8. MaROP5g Overexpression Increased the Expression of Salt Overly Sensitive (SOS)-Pathway and Ca^2+^-Sensing Genes

**Figure 7:**
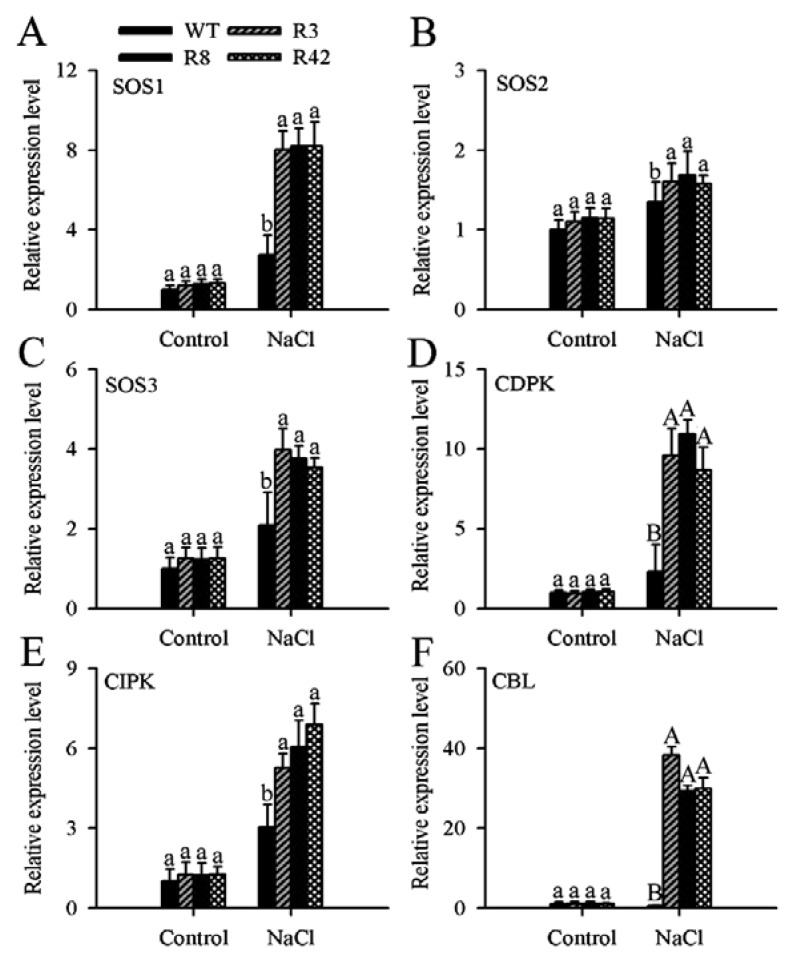
Expression of Salt Overly Sensitive (SOS)- and calcium-signaling genes in wild-type and *A. thaliana* overexpressing *MaROP5g*.WT: Wild-type. R3, R8, R42: *MaROP5g* transgenic plants. (**A**–**F**) Expression patterns of *SOS1*, *SOS2*, *SOS3*, *CDPK*, *CIPK*, and *CBL* genes in wild-type and transgenic roots under normal conditions and salt treatment. ANOVA was used to compare the significance of differences, using Student’s *t* tests in the comparison between control and NaCl treatment. Data are presented as means ± standard deviations of *n* = 3 biological replicates. Different lowercase letters above bars indicate significant differences at *p* < 0.05, and different uppercase letters above bars indicate extremely significant differences at *p* < 0.01.

To gain an in-depth understanding of *MaROP5g* function in response to salt stress, we measured the expression of three Salt Overly Sensitive (SOS)-pathway genes (namely *SOS1*, *SOS2*, and *SOS3*) and several genes encoding calcium-signaling pathway proteins, including calcineurin B-like (CBL) proteins, CBL-interacting protein kinases (CIPKs), and calcium-dependent protein kinases (CDPKs) [39], in both WT and *MaROP5g*-overexpressing *A. thaliana* (Figure 7). Under standard growth conditions, we observed no significant differences in the transcription levels of the tested genes in the transgenic lines as compared to those in WT plants. However, under salt stress, the gene expression levels of *SOS1*, *SOS2*, *SOS3*, *CBL*, *CIPK*, and *CDPK* were higher in the transgenic lines as compared to those in WT. This indicated that *MaROP5g* overexpression in response to salt stress led to the up-regulation of both SOS-pathway genes and calcium-signaling pathway genes.

## 3. Discussion

Despite its economic and social importance, research on banana plants has generally been slower relative to many other crops, especially with respect to the abiotic stress responses [31,32]. ROP is an important molecular switch involved in plant signal transduction processes, which has been suggested to play crucial roles in the regulation of the environmental stress responses in numerous plant species [8,11,21,40]. We have identified 17 *MaROPs* by searching the *M. acuminata* genome, which were classified into three groups (II–IV), following the nomenclature derived for *OsROPs* [18]. Of the 17 *MaROPs*, none was categorized into Group I, congruent with *ROPs* in other higher plants, such as *O. sativa* [18], *Z. mays* [16], *Medicago truncatula* [7], and *N. tabacum* [7]. The recovered phylogenetic relationships were further supported by our analyses of gene structure and conserved motifs. The *MaROP* genomic sequences in Groups II–IV were found to contain 6–8 exons and 6–7 introns. Similar structural features have been observed in *ROPs* of other plant species, including *N. tabacum* [23], *A. thaliana* [15], and *M. truncatula* [7]. Moreover, all of the identified MaROPs had the typical RhoGEF domain (PF00621), and MaROP proteins within each group shared similarly conserved motifs (Figure 2), which is consistent with the observations in *M. truncatula* [7].

Bananas are extremely sensitive to abiotic stress and can suffer severe losses in yield and quality when exposed to cold, salt, or drought conditions [36]. We found that 82% of the 17 *MaROPs* showed transcriptional changes following cold, salt, and osmotic stress treatments (Figure 3). Interestingly, except the high expression genes (*MaROP-3b*, *-5a*, *-5c*, *-5f*, *-5g*, and *-6*) under three stress treatments, *MaROP-3a* and *-5h* are significantly downregulated in the presence of stress in BX and FJ compared to control. To our knowledge, this is the first report showing that banana *MaROPs* exhibit extensive and diverse responses to abiotic stressors. The induction of *ROP* expression by cold, salt, and drought has previously been reported in other plants, such as *Malus*× *domestica* Borkh [21], *A. thaliana* [22], and *N. tabacum* [23].

It is particularly important to note that the expression of *MaROP5g* among the 17 *MaROP* genes was most highly induced following salt stress treatment across both banana genotypes tested (Figure 3 and Figure 4A–D), which may imply its positive role in mediating banana’s response to salt stress. Further, although *MaROP5g* expression in roots of both BX and FJ genotypes can be induced by salt stress treatment, BX showed more sensitivity than FJ under salt stress treatment. This may suggest that BX, with its genome constitution as AAA, is more sensitive to salt treatment in comparison to the B-genome-containing genotype FJ. Such an observation is consistent with previous studies that FJ, with AAB genotypes, exhibited higher tolerance to abiotic stresses relative to BX [27].The MaROP5g protein was located on the plasma membrane (Figure 4E), consistent with *A. thaliana* ROP2 [41] and rice OsRac5 [18]. To better understand the function of *MaROP5g* during salt stress, we generated a number of *MaROP5g*-overexpressing transgenic *A. thaliana* lines. Under salt stress, the transgenic seedlings and adult plants exhibited a higher survival rate and increased root length as compared to WT (Figure 5), suggesting that *MaROP5g* overexpression might contribute to the maintenance of a healthy growth status, through the improvement of root development and distributions [24,25], and hence enhance salt stress tolerance.

As cell membranes are one of the primary targets of various environmental stresses, MDA is commonly used as an index of oxidative damages [36], and IL is used as an important indicator of membrane injury in plant research [36]. MDA content and IL were measured to assess the role of *MaROP5g* overexpression in reducing membrane injury under salt conditions. *MaROP5g* overexpression resulted in decreased IL and MDA content relative to WT, indicating that *MaROP5g*-overexpressing plants may experience less membrane injury and maintain a healthy physiological status under salt conditions.

In plants, high K^+^ and low Na^+^ concentrations are beneficial for the maintenance of physiological processes under salt stress [42]. In recent years, a high cytosolic K^+^/Na^+^ ratio has become an accepted marker of salinity tolerance [38]. Previous studies have reported that the expression of *MfARL1* resulted in a reduced Na^+^/K^+^ ratio in transgenic *A. thaliana* as compared to WT, due to a lower accumulation of Na^+^ [24]. Under salt stress, *MaROP5g* overexpression decreased the accumulation of cellular Na^+^, increased the Ca^2+^ concentration, and improved the K^+^/Na^+^ ratio in transgenic *A. thaliana* as compared to WT plants (Figure 6). Therefore, the increased salt stress tolerance conferred by *MaROP5g* overexpression may be due not only to the decreased Na^+^ accumulation, but also to the increased Ca^2+^ concentration in transgenic lines as compared to WT.

Many different ion transporters and channel proteins, such as SOS, CDPK, CBL, and CIPK, play crucial roles in maintaining ion homeostasis during salt stress [39,42,43]. For example, under salt stress, the SOS1 and SOS2 proteins regulate Na^+^/K^+^ homeostasis in *A. thaliana*; once the calcium binding protein SOS3 senses an increase in cytosolic calcium concentration, the SOS3–SOS2 protein kinase complex activates the SOS1 ion transporter [42,43]. In addition, calcium (Ca^2+^), as a second messenger, plays an important role in salt stress processes [39,44]. Ca^2+^ increase can be decoded and recognized by Ca^2+^ sensors, including CBLs, CIPKs, and CDPKs [44]. CBLs recognize the increase in cytosolic Ca^2+^ concentration triggered by Na^+^ accumulation [39]. CIPKs and CDPKs may unify and coordinate ionic homeostasis at the cellular and organismal level [44]. We examined the expression of these SOS- and calcium-signaling pathway genes in the *MaROP5g*-overexpressing transgenic *A. thaliana* seedlings in relation to WT seedlings. Following salt treatment, SOS- and calcium-signaling pathway genes were a significant expression in the transgenic seedlings as compared to WT seedlings. This suggested that the *MaROP5g-*overexpressing transgenic plants were more responsive to SOS- and calcium-signaling compared to WT plants, implying that *MaROP5g*-overexpressing plants had improved Na^+^ and Ca^2+^ ionic homeostasis under salt stress conditions.

## 4. Experimental Section

### 4.1. Plant Materials

BaXi Jiao (BX; *M. acuminata* cv. Cavendish; AAA group) is a triploid banana cultivar that is of high yield and high quality and can be stored for an extended period of time [31,32]. Fen Jiao (FJ; *M. acuminata*; group AAB), another triploid banana cultivar, has good flavor, rapid ripening, and a high tolerance for abiotic stress [31,32]. Both of these banana cultivars were planted and maintained at the banana plantation of the Chinese Academy of Tropical Agricultural Sciences (Danzhou, Hainan, China; 19°11′–19°52′ N, 108°56′–109°46′ E). All of the banana plants were grown in 70% relative humidity at 28 °C, with 200 μmol·m^−2^·s^−1^ light in cycles of 16 h light/8 h dark (Sylvania GRO LUX fluorescent lamps; Utrecht, The Netherlands).

For the salt and drought-simulation experiments, five-leaf stage banana plants of both cultivars were irrigated with 300 mmol·L^−1^ NaCl or 200 mmol·L^−1^ mannitol, respectively, for 7 days, as previously described by Hu et al. [27]. For the cold experiments, five-leaf stage banana plants of both cultivars were subjected to 4 °C for 22 h, as previously described by Hu et al. [27]. For the control experiments, five-leaf stage banana plants of both cultivars were irrigated with equal volume water with the stress groups at 28 °C. After the completion of each treatment, we harvested and immediately froze in liquid nitrogen the leaves and root systems of each plant, which were stored at −80 °C. Twelve five-leaf stage banana plants and three biological replicates were performed for each treatment.

### 4.2. Identification and Phylogeny of the MaROP Gene Family

The banana ROP protein sequences were downloaded from the DH-Pahang genome database (*M. acuminata*, A-genome, 2*n* = 22) (available online: http://banana-genome.cirad.fr) [33]. ROP amino acid sequences from *A. thaliana* (AtROPs) and *O. sativa* (OsROPs) were downloaded from the TAIR (The Arabidopsis Information Resource) (available online: http://www.arabidopsis.org) and RGAP (Rice Genome Annotation Project) (available online: http://rice.plantbiology.msu.edu) databases, respectively. HMMER (available online: http://hmmer.org) was used to predict conserved RhoGEF domains (PF00621; available online: http://pfam.sanger.ac.uk) in the ROP proteins [34]. The basic local alignment search tool (BLAST) (available online: http://www.ncbi.nlm.nih.gov/BLAST/) was used to identify putative MaROPs, based on the sequences of the AtROPs and OsROPs [35]. The conserved domains of the putative MaROPs were identified with the Conserved Domain Database (available online: http://www.ncbi.nlm.nih.gov/cdd) and validated with PFAM (available online: http://pfam.sanger.ac.uk) [45,46,47]. Identity numbers of all of the putative MaROPs that we have identified was presented in Table S1. All of the MaROP, AtROP, and OsROP sequences were aligned with Multiple Sequence Alignment (MUSCLE), and a bootstrapped ML phylogenetic tree (1000 replicates) was constructed in MEGA 5.2 (available online: http://www.megasoftware.net/) using this alignment [48].

### 4.3. Protein Properties and Gene Structure

We predicted the molecular masses and isoelectric points of the putative MaROP proteins with the Expert Protein Analysis System database (available online: http://expasy.org/) [49]. We constructed a bootstrapped ML phylogenetic tree (1000 replicates) in MEGA 5.2 software by aligning all MaROP sequences with MUSCLE [47]. MaROP protein motifs were identified with Multiple Em for Motif Elicitation (available online: http://meme-suite.org) and annotated using InterProScan (available online: http://www.ebi.ac.uk/Tools/pfa/iprscan) [50,51]. Structural features of the *MaROP* genes were identified with Gene Structure Display Server (available online: http://gsds.cbi.pku.edu.cn) by comparing the nucleotide sequences to predicted coding regions for all *MaROPs* [52]. *MaROP* promoter sequences were obtained from the banana genome database (available online: http://banana-genome.cirad.fr) [33]. Based on fragments 2000 bp upstream of each *MaROP*, a transcription start site was predicted with the Berkeley *Drosophila* Genome Project database (available online: http://www.fruitfly.org/seq_tools/promoter.html) and the *cis*-acting elements were predicted with PlantCARE (available online: http://bioinformatics.psb.ugent.be/webtools/plantcare/html) [53,54].

### 4.4. Transcriptomic Analysis

We isolated total RNA from the leaf tissues of the banana seedlings subjected to each of the three treatments (salt, osmosis, and cold) and control (no stress conditions), which were constructed into respective cDNA libraries [31,32]. Deep paired-end sequencing was performed with an Illumina GAII according to manufacturer’s instructions. There are two replicates for each sample. The sequencing depth was 5.34X on average. Adaper sequences in the raw reads were removed using FASTX-tookit (Illlumina, San Diego, CA, USA). Using Tophat v.2.0.10, clean reads were mapped to the DH-Pahang genome [33]. The transcriptome assemblies were performed by Cufflinks [27]. Gene expression levels were calculated as fragments per kilobase of exon per million fragments mapped (FPKM). DEGseq was used to identify differently expressed genes [55]. Under three stress conditions, the expression level of each gene was compared with the control. A heat map was created based on the FPKM value of the *MaROPs*, compared to the control by MeV 4.9.0 software (available online: https://sourceforge.net/projects/mev-tm4/).

### 4.5. QRT-PCR Analysis

The gene expression of *MaROPs* in response to cold, salt, and osmotic (drought) stress was measured with qPCR, using a SYBR Premix ExTaq kit (TaKaRa, Shiga, Japan) on a Stratagene Mx3000P detection system (Stratagene, San Diego, CA, USA). The primer pairs with high specificity and efficiency were selected based on their melting curve and on agarose gel electrophoresis (Table S5). The amplification efficiencies of the primer pairs chosen ranged from 0.9 to 1.1. *MaActin* (EF672732) and *MaUBQ2* (HQ853254) were used as the internal controls. The expression levels of *MaROP* relative to *MaActin* and *MaUBQ2* were calculated with the 2^−ΔΔ*C*T^ method [56]. Three biological replicates for each sample were performed.

### 4.6. Full-Length cDNA of MaROP5g and Gene Expression During Salt Treatment

Based on our RNA-seq results, we selected the *ROP* gene *MaROP5g* for further analysis. The entire coding region of *MaROP5g* was amplified with PCR, using single-stranded cDNA obtained from the roots of banana plants subjected to salt stress as the source template, using a specific primer pair (5′-gcaccatggagatgagcgcgtcgaggt-3′ and 5′-gcgactagtcaatatggagcaacctttc-3′). The resulting *MaROP5g* fragment was verified with DNAMAN (available online: http://www.lynnon.com/) and compared to the genome database of DH-Pahang using BLAST [33,35].

Twelve ex vitro banana plants with uniform growth at the five-leaf stage were selected and divided into four groups for salt treatments, which were irrigated with half-strength Hoagland solution, supplemented with 300 mM NaCl for 0, 2, 4, or 6 h (*n* = 4 per time period), as previously described by Xu et al. [36]. Samples were frozen individually in liquid nitrogen and stored at −70 °C. Compared to the expression of 0 h in BX, the relative expression level of *MaROP5g* at 2, 4, and 6 h under salt stress in BX was calculated. Compared to the expression of 0 h in FJ, the relative expression level of *MaROP5g* at 2, 4, and 6 h under salt stress in FJ banana plants was calculated.

### 4.7. Subcellular Localization of MaROP5g

The ORF of *MaROP5g* was digested with the restriction enzymes *Nco* I and *Spe* I and inserted into a pCAMBIA1304-GFP expression vector to generate a MaROP5g-GFP fusion protein, under the control of the cauliflower mosaic virus (CaMV) 35S promoter. The recombinant pCAMBIA1304-MaROP5g-GFP plasmid was transferred to *Agrobacterium tumefaciens* strain LBA4404, and used to transform *A. thaliana* through a floral-dip method [57]. Root tips (3–5 mm) of *A. thaliana* seedlings (5-day old) with a stable expression of MaROP5g-GFP were incubated in 1 mL 1/2 Murashige and Skoog (MS) medium containing 10 μg FM4-64 (Invitrogen, Carlsbad, CA, USA) for 5 min at 25 °C, according to the Riqal et al. [58] methods. The GFP (488 nm emission filter) and FM4-64 (543 nm emission filter) signals were visualized using confocal laser scanning microscopy (CLSM; Nikon, A1, Tokyo, Japan). According to the Protein Subcellular Localization Prediction Tool (PSORT) software (available online: www.genscript.com/psort.html) prediction, the XXRR-like motif in the N-terminus of MaROP5g protein was identified as a membrane retention.

### 4.8. Plant Transformation and Generation of Transgenic Plants

The ORF of *MaROP5g* was digested with the restriction enzymes *Nco* I and *Spe* I, and inserted into a pCAMBIA1304 vector. The recombinant pCAMBIA1304-MaROP5g plasmid was transferred to *A. tumefaciens* strain LBA4404 [57]. Transgenic *A. thaliana* plants were generated using the floral dip-mediated infiltration method [57]. Seeds from T_0_ transgenic plants were plated in kanamycin selection medium (50 mg·L^−1^). The homozygous T_3_ lines were used for further functional investigation of *MaROP5g*.

### 4.9. Southern Blot Analyses

Genomic DNA isolated from the T_3_ generation kanamycin-resistant transgenic lines was digested with the *EcoR* I restriction enzyme. A 436 bp region of *MaROP5g* was amplified by PCR, using a pair of specific oligo primers (5′-gtggtggatggtaacacagtta-3′ and 5′-aacctttctgttgcttttttttc-3′). Based on this sequence, we prepared a hybridization probe for use with DIG-dUTP (Roche Applied Science, Mannheim, Germany), following the manufacturer’s instructions. After hybridization, the HyBond N^+^ membrane (Amersham) was washed and exposed to X-ray film (Kodak BioMax MS, Kodak Eastman, Rochester, NY, USA), following the method described by Miao et al. [59].

### 4.10. Salt Stress Treatments in WT and Transgenic Plants

The seeds of both transgenic and WT *A. thaliana* (Columbia ecotype; control) were first vernalized for 2 days at 4 °C in the dark, and surface sterilization in 75% ethanol for 10 min, prior to germination on half-strength MS medium or directly in soil. These *A. thaliana* plants were maintained at 22 °C with 70% humidity and a 16 h light/8 h dark cycle (Sylvania GRO LUX fluorescent lamps; Utrecht, The Netherlands). To analyze *A. thaliana* phenotypes in early seedlings under normal conditions, four day-old seedlings were transferred to 1/2 MS medium for 15 days, photos were taken, and root lengths were measured. To test salt stress tolerance in early seedlings, four day-old seedlings were transferred to either 1/2 MS or 1/2 MS supplemented with 100–200 mM NaCl for 15 days, after which photos were taken and the root length was measured. To test salt stress tolerance in adult plants, 4-week-old *A. thaliana* plants were irrigated with 350 mM NaCl for 15 days, and then photos were taken and survival rates were assessed (leaves fall and roots rot were identified as death). To measure the expression of SOS- and calcium-signal pathway genes in the WT and transgenic lines, 15-day-old seedlings were transferred to 1/2 MS supplemented with 350 mM NaCl for up to 10 h. Whole leaves were used to quantify relative gene expression, using qRT-PCR (see Table S5 for primer sequences).

### 4.11. Measurement of IL and MDA Content

Four-week-old *A. thaliana* plants were irrigated with 350 mM NaCl for 15 days and leaf samples were collected to examine IL and MDA. IL was detected according to the method described by Xu et al. [36]. Leaf samples were cut into strips and incubated in 10 mL of distilled water at 25 °C for 8 h. The initial conductivity (C1) was determined with a conductivity meter (DDBJ-350). The samples were then boiled for 10 min to yield complete IL. After cooling down, the electrolyte conductivity (C2) was measured. IL was calculated according to the equation: IL (%) = C1/C2:100. MDA content was measured according to the thiobarbituric acid colorimetric method, as described by Xu et al. [36].

### 4.12. Ca^2+^, Na^+^, and K^+^ Concentrations

We irrigated 4-week-old WT and transgenic plants with 350 mM NaCl for 15 days. We then collected the plant roots and washed them with ultrapure water. Plant roots were heated to 105 °C for 10 min and then dried at 80 °C for 48 h. We dissolved 50 mg of each dried sample in 6 mL nitric acid and 2 mL H_2_O_2_ (30%), and then heated the solution to 180 °C for 15 min. The digested samples were diluted to a total volume of 50 mL with ultrapure water, transferred to clean tubes, and analyzed with atomic absorption spectroscopy (Analyst400, Perkin Elmer, Waltham, MA, USA).

### 4.13. Statistical Analysis

Three biological replicates for each sample were performed. Statistical analyses were performed using SPSS 19.0 (Chicago, IL, USA). We used analyses of variance (ANOVAs) to compare the significance of differences based on Dunnett’s tests or Student’s *t* tests. Specifically, Dunnett’s tests were used to compare between WT and each overexpression line, while Student’s *t* tests were used to compare between control and NaCl treatment. *p* < 0.05 was considered a significant level and *p* < 0.01 an extremely significant level.

## 5. Conclusions

In this study, for the first time for banana, we identified 17 *MaROP* genes in the *M. acuminata* genome, and classified these genes into three groups (II–IV) based on phylogeny, gene structure, and conserved protein motifs. The expression patterns of the *MaROP* genes in response to abiotic stress as reported here may shed light on the possible involvement of these genes in the regulation of abiotic stress signaling pathways. Of particular interest was *MaROP5g*, the overexpression of which increased the plant’s tolerance for salt stress not only by maintaining a healthy growth status, but also by reducing membrane injury and improving ion distribution. Our results lay a foundation for genetic improvements in the banana plant, increasing resistance to various abiotic stressors, particularly salt. It is necessary to point out that these conclusions were drawn from the heterologous expression of banana *MaROP5g* in *A. thaliana* as a model plant system, which may or may not be valid in other plant systems. Further studies are required to characterize the function of *MaROP5g* in banana.

## Supplementary Materials

Supplementary materials can be found at http://www.mdpi.com/1422-0067/19/10/3108/s1.

## References

[1] Takai Y., Sasaki T., Matozaki T. (2001). Small GTP-binding proteins. Physiol. Rev..

[2] Ono E., Wong H.L., Kawasaki T., Hasegawa M., Kodama O., Shimamoto K. (2001). Essential role of the small GTPase Rac in disease resistance of rice. Proc. Natl. Acad. Sci. USA.

[3] Rocha N., Payne F., Huang-Doran I., Sleigh A., Fawcett K., Adams C., Stears A., Saudek V., O’Rahilly S., Barroso I., Semple R.K. (2017). The metabolic syndrome-associated small G protein ARL15 plays a role in adipocyte differentiation and adiponectin secretion. Sci. Rep..

[4] Eliáš M., Klimeš V. (2012). Rho GTPases: Deciphering the evolutionary history of a complex protein family. Methods Mol. Biol..

[5] Feiquelman G., Fu Y., Yalovsky S. (2018). RopGTPases structure-function and signaling pathyways. Plant Physiol..

[6] Winge P., Brembu T., Bones A.M. (1997). Cloning and characterization of rac-like cDNAs from *Arabidopsis thaliana*. Plant Mol. Biol..

[7] Liu W., Chen A.M., Luo L., Sun J., Cao L.P., Yu G.Q., Zhu J.B., Wang Y.Z. (2010). Characterization and expression analysis of *Medicago truncatula* ROP GTPase family during the early stage of symbiosis. J. Integr. Plant Biol..

[8] Zheng Z.L., Yang Z. (2000). The rop GTPase switch turns on polar growth in pollen. Trends Plant Sci..

[9] Abbal P., Pradal M., Sauvage F.X., Chatelet P., Paillard S., Canaguier A., Adam-Blondon A.F., Tesniere C. (2007). Molecular characterization and expression analysis of the Rop GTPase family in *Vitis vinifera*. J. Exp. Bot..

[10] Zhang Y., McCormick S. (2010). The regulation of vesicle trafficking by small GTPases and phospholipids during pollen tube growth. Sex Plant Reprod..

[11] Huang J.B., Liu H., Chen M., Li X., Wang M., Yang Y., Wang C., Huang J., Liu G., Liu Y. (2014). Rop3 GTPase contributes to polar auxin transport and auxin responses and is important for embryogenesis and seedling growth in Arabidopsis. Plant Cell.

[12] Poraty-Gavra L., Zimmermann P., Haigis S., Bednarek P., Hazak O., Stelmakh O.R., Sadot E., Schulze-Lefert P., Gruissem W., Yalovsky S. (2013). The Arabidopsis Rho of plants GTPase AtROP6 functions in developmental and pathogen response pathways. Plant Physiol..

[13] Ma Q.H., Zhu H.H., Han J.Q. (2017). Wheat ROP proteins modulate defense response through lignin metabolism. Plant Sci..

[14] Yang Z., Watson J.C. (1993). Molecular cloning and characterization of rho, a ras-related small GTP-binding protein from the garden pea. Proc. Natl. Acad. Sci. USA.

[15] Li H., Shen J.J., Zheng Z.L., Lin Y.K., Yang Z.B. (2001). The Rop GTPase switch controls multiple developmental processes in Arabidopsis. Plant Physiol..

[16] Christensen T.M., Vejlupkova Z., Sharma Y.K., Arthur K.M., Spatafora J.W., Albright C.A., Meeley R.B., Duvick J.P., Quatrano R.S., Fowler J.E. (2003). Conserved subgroups and developmental regulation in the monocot *rop* gene family. Plant Physiol..

[17] Chan J., Pauls P.K. (2007). Brassica napus Rop GTPases and their expression in microspore cultures. Planta.

[18] Chen L., Shiotani K., Togashi T., Miki D., Aoyama M., Wong H.L., Kawasaki T., Shimamoto K. (2010). Analysis of the Rac/Rop small GTPase family in rice: Expression, subcellular localization and role in disease resistance. Plant Cell Physiol..

[19] Qin Y.X., Huang Y.C., Fang Y.J., Qi J.Y., Tang C.R. (2014). Molecular characterization and expression analysis of the small GTPase ROP members expressed in laticifers of the rubber tree (*Hevea brasiliensis*). Plant Physiol. Biochem..

[20] Liang Q.X., Cao G.Q., Zhao S.P., Huang Q.C., Ying F.Q., Chen W. (2015). Analysis of ROP signaling in the leaf epidermis of mutant tomato with low-energy ion beam. Genet. Mol. Res..

[21] Zermiani M., Zonin E., Nonis A., Begheldo M., Ceccato L., Vezzaro A., Baldan B., Trentin A., Masi A., Fadanelli L. (2015). Ethylene negatively regulates transcript abundance of ROP-GAP rheostat-encoding genes and affects apoplastic reactive oxygen species homeostasis in epicarps of cold stored apple fruits. J. Exp. Bot..

[22] Li Z., Kang J., Sui N., Liu D. (2012). ROP11 GTPase is a negative regulator of multiple ABA responses in Arabidopsis. J. Integr. Plant Biol..

[23] Cao Y., Li Z., Chen T., Zhang Z., Zhang J., Chen S. (2008). Overexpression of a tobacco small G protein gene NtRop1 causes salt sensitivity and hydrogen peroxide production in transgenic plants. Sci. China C Life Sci..

[24] Wang T.Z., Xia X.Z., Zhao M.G., Tian Q.Y., Zhang W.H. (2013). Expression of a Medicago falcata small GTPase gene, MfARL1 enhanced tolerance to salt stress in *Arabidopsis thaliana*. Plant Physiol. Biochem..

[25] Li C., Lu H., Li W., Yuan M., Fu Y. (2017). A ROP2-RIC1 pathway fine-tunes microtubule reorganization for salt tolerance in Arabidopsis. Plant Cell Environ..

[26] Paul J.Y., Khanna H., Kleidon J., Hoang P., Geijskes J., Daniells J., Zaplin E., Rosenberg Y., James A., Mlalazi B. (2017). Golden bananas in the field: Elevated fruit pro-vitamin A from the expression of a single banana transgene. Plant Biotechnol. J..

[27] Hu W., Wang L., Tie W., Yan Y., Ding Z., Liu J., Li M., Peng M., Xu B., Jin Z. (2016). Genome-wide analyses of the bZIP family reveal their involvement in the development, ripening and abiotic stress response in banana. Sci. Rep..

[28] Sreedharan S., Shekhawat U.K., Ganapathi T.R. (2015). Constitutive and stress-inducible overexpression of a native aquaporin gene (MusaPIP2;6) in transgenic banana plants signals its pivotal role in salt tolerance. Plant Mol. Biol..

[29] Lee W.S., Gudimella R., Wong G.R., Tammi M.T., Khalid N., Harikrishna J.A. (2015). Transcripts and MicroRNAs responding to salt stress in *Musa acuminata* Colla (AAA Group) cv. Berangan roots. PLoS ONE.

[30] Hu W., Yan Y., Shi H., Miao H., Tie W., Ding Z., Wu C., Liu Y., Wang J., Xu B., Jin Z. (2017). The core regulatory network of the abscisic acid pathway in banana: Genome-wide identification and expression analyses during development, ripening, and abiotic stress. BMC Plant Biol..

[31] Miao H.X., Sun P.G., Liu Q., Miao Y.L., Liu J.H., Zhang K.X., Hu W., Zhang J.B., Wang J.Y., Wang Z. (2017). Genome-wide analyses of SWEET family proteins reveal involvement in fruit development and abiotic/biotic stress responses in banana. Sci. Rep..

[32] Miao H.X., Sun P.G., Liu Q., Liu J.H., Xu B.Y., Jin Z.Q. (2017). The AGPase family proteins in banana: Genome-wide identification, phylogeny, and expression analyses reveal their involvement in the development, ripening, and abiotic/biotic stress responses. Int. J. Mol. Sci..

[33] D’Hont A., Denoeud F., Aury J.M., Baurens F.C., Carreel F., Garsmeur O., Noel B., Bocs S., Droc G., Rouard M. (2012). The banana (*Musa acuminata*) genome and the evolution of monocotyledonous plants. Nature.

[34] Eddy S.R. (2009). A new generation of homology search tools based on probabilistic inference. Genome Inform..

[35] Altschul S.F., Gish W., Miller W., Myers E.W., Lipman D.J. (1990). Basic local alignment search tool. J. Mol. Biol..

[36] Xu Y., Hu W., Liu J.H., Zhang J.B., Jia C.H., Miao H.X., Xu B.Y., Jin Z.Q. (2014). A banana aquaporin gene, MaPIP1; 1, is involved in tolerance to drought and salt stresses. BMC Plant Biol..

[37] Han S., Wang C.W., Wang W.L., Jiang J. (2014). Mitogen-activated protein kinase 6 controls root growth in Arabidopsis by modulating Ca2+-based Na+ flux in root cell under salt stress. J. Plant Physiol..

[38] Ruiz-Lozano J.M., Porcel R., Azcón C., Aroca R. (2012). Regulation by arbuscular mycorrhizae of the integrated physiological response to salinity in plants: New challenges in physiological and molecular studies. J. Exp. Bot..

[39] Miranda R.S., Alvarez-Pizarro J.C., Costa J.H., Paula S.O., Pirsco J.T., Gomes-Filho E. (2017). Putative role of glutamine in the activation of CBL/CIPK signaling pathways during salt stress in sorghum. Plant Signal. Behav..

[40] Hoefle C., Huesmann C., Schultheiss H., Bornke F., Hensel G., Kumlehn J., Huckelhoven R. (2011). A barley ROP GTPase ACTIVATING PROTEIN associates with microtubules and regulates entry of the barley powdery mildew fungus into leaf epidermal cells. Plant Cell.

[41] Jeon B.W., Hwang J.U., Hwang Y., Song W.Y., Fu Y., Gu Y., Bao F., Cho D., Kwak J.M., Yang Z. (2008). The Arabidopsis small G protein ROP2 is activated by light in guard cells and inhibits light-induced stomatal opening. Plant Cell.

[42] Shi H., Ishitani M., Kim C., Zhu J.K. (2000). The *Arabidopsis thaliana* salt tolerance gene *SOS1* encodes a putative Na^+^/H^+^ antiporter. Proc. Natl. Acad. Sci. USA.

[43] Liu J., Ishitani M., Halfter U., Kim C.S., Zhu J.K. (2000). The *Arabidopsis thaliana SOS2* gene encodes a protein kinase that is required for salt tolerance. Proc. Natl. Acad. Sci. USA.

[44] Köster P., Wallrad L., Edel K.H., Faisal M., Alatar A.A., Kudla J. (2018). The battle of two ions: Ca^2+^ signaling against Na^+^ stress. Plant Biol..

[45] Marchler-Bauer A., Bo Y., Han L., He J., Lanczycki C.J., Lu S., Lu S., Chitsaz F., Derbyshire M.K., Geer R.C. (2017). CDD/SPARCLE: Functional classification of proteins via subfamily domain architectures. Nucleic Acids Res..

[46] Finn R.D., Coggill P., Eberhardt R.Y., Eddy S.R., Mistry J., Mitchell A.L., Potter S.C., Punta M., Qureshi M., Sangrador-Vegas A. (2016). The Pfam protein families database: Towards a more sustainable future. Nucleic Acids Res..

[47] Larkin M.A., Blackshields G., Brown N.P., Chenna R., Mcgettigan P.A., Mcwilliam H., Valentin F., Wallace I.M., Wilm A., Lopez R. (2007). Clustal W and Clustal X version 2.0. Bionformatics.

[48] Tamura K., Peterson D., Peterson N., Stecher G., Nei M., Kumar S. (2011). MEGA5: Molecular evolutionary genetic analysis using maximum likelihood, evolutionary distance, and maximum parsimony methods. Mol. Biol. Evol..

[49] Gasteiger E., Gattiker A., Hoogland C., Ivanyi I., Appel R.D., Bairoch A. (2003). ExPASy: The proteomics server for in-depth protein knowledge and analysis. Nucleic Acids Res..

[50] Bailey T.L., Boden M., Buske F.A., Frith M., Grant C.E., Clementi L., Ren J., Li W.W., Noble W.S. (2009). MEME SUITE: Tools for motif discovery and searching. Nucleic Acids Res..

[51] Jones P., Binns D., Chang H.Y., Fraser M., Li W., McAnulla C., McWilliam H., Maslen J., Mitchell A., Nuka G. (2014). InterProScan 5: Genome-scale protein function classification. Bioinformatics.

[52] Hu B., Jin J., Guo A.Y., Zhang H., Luo J., Gao G. (2015). GSDS 2.0: An upgraded gene feature visualization server. Bioinformatics.

[53] Celniker S.E., Wheeler D.A., Kronmiller B., Carlson J.W., Haipern A., Patel S., Adams M., Champe M., Dugan S.P., Frise E. (2002). Finishing a whole-genome shotgun: Release 3 of the *Drosophila melanogaster* euchromatic genome sequence. Genome Biol..

[54] Lescot M., Déhais P., Thijs G., Marchal K., Moreau Y., Peer Y.V., Rouzé P., Rombauts S. (2002). PlantCARE, a database of plant *cis*-acting regulatory elements and a portal to tools for in silico analysis of promoter sequences. Nucleic Acids Res..

[55] Wang L., Feng Z., Wang X., Wang X., Zhang X. (2010). DEGseq: An R package for identifying differentially expressed genes from RNA-seq data. Bioinformatics.

[56] Livak K.J., Schmittgen T.D. (2001). Analysis of relative gene expression data using real-time quantitative PCR and the 2^−ΔΔ*C*T^ Method. Methods.

[57] Clough S.J., Bent A.F. (1998). Floral dip: A simplified method for *Agrobacterium*-mediated transformation of *Arabidopsis thaliana*. Plant J..

[58] Riqal A., Doyle S.M., Robert S. (2015). Live cell imaging of FM4-64, a tool for tracing the endocytic pathways in Arabidopsis root cells. Methods Mol. Biol..

[59] Miao H.X., Qin Y.H., Teixeira da Silva J.A., Ye Z.X., Hu G.B. (2011). Cloning and expression analysis of *S-RNase* homologous gene in *Citrus reticulata* Blanco cv. Wuzishatangju. Plant Sci..

