# Supplementary material for: Overexpression of a Novel ROP Gene from the Banana (MaROP5g) Confers Increased Salt Stress Tolerance
Source: Int J Mol Sci. 2018 Oct 11;19(10):3108. doi: 10.3390/ijms19103108 (PMC6213407; doi:10.3390/ijms19103108)
Supplement: Supplementary file 1 [file ijms-19-03108-s001.zip › Figure S1&S2.pdf]

>MaRop5g

ATGAGCGCGTCGAGGTTTATAAAGTGCGTCACGGTGGGGGACGGTGCCGTCGGCAAGACTTGCATGCTTAT
CTCCTACACCAGCAACACTTTCCCCACGGATTATGTTCCTACCGTTTTTGACAATTTCAGCGCAAATGTAGTG
GTGGATGGTAACACAGTTAACTTAGGTTTGTGGGATACTGCAGGCCAAGAGGATTACAATAGATTAAGACCT
TTGAGCTACCGTGGTGCAGATGTTTTCTTGCTTGCTTTCTCTCTCATTAGTAAAGCCAGCTATGAAAATGTTGC
CAAGAAGTGGATTCCTGAGCTAAGGCACTATGCACCTGGTGTGCCCATAATTCTTGTTGGAACAAAGCTTGA
TCTTCGGGATGAGCAGCAGTTCTTTATAGATCACCCAGGTGCTGTTCCCATAAGTACCGCTCAGGGGGAGGA
GCTGCGGAAGCAAATAGGTGCTGCTTCGTACATAGAGTGCAGTTCAAAGACTCAGCAAAATGTCAAGGCGG
TATTTGATGCGGCCATCAAGGTCGTGCTTCAGCCACCCAAGCAGAAGAAGAAAAAAAAGCAACAGAAAGG
TTGCTCCATATTGTGA

MSASRFIKCVTVGDGAVGKTCMLISYTSNTFPTDYVPTVFDNFSANVVVDGNTVNLGLWD
TAGQEDYNRLRPLSYRGADVFLLAFSLISKASYENVAKKWIPELRHYAPGVPIILVGTKL
DLRDEQQFFIDHPGAVPISTAQGEELRKQIGAASYIECSSKTQQNVKAVFDAAIKVVLQP
PKQKKKKKQQKGCSIL-

Figure S1. Full-length cDNA and amino acid sequence of *MaROP5g* from banana plants.

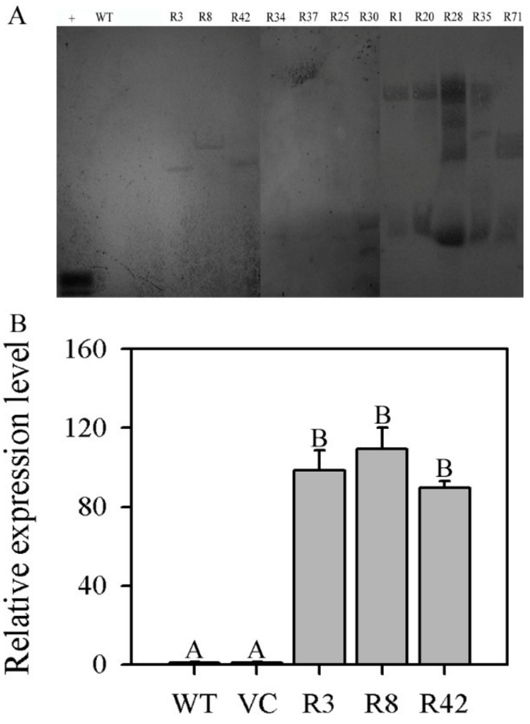

Figure S2. Southern blot analysis and qRT-PCR analysis of *MaROP5g* transgenic plants in *A. thaliana*. (**A**) Southern blot; (**B**) qRT-PCR. +: positive control. WT: wild-type. R3, R8, R42, R34, R37, R25, R30, R1, R20, R28, R35, R71: *MaROP5g* transgenic plants.
